# Imaging Features and Patterns of Metastasis in Non-Small Cell Lung Cancer with *RET* Rearrangements

**DOI:** 10.3390/cancers12030693

**Published:** 2020-03-15

**Authors:** Subba R. Digumarthy, Dexter P. Mendoza, Jessica J. Lin, Marguerite Rooney, Andrew Do, Emily Chin, Beow Y. Yeap, Alice T. Shaw, Justin F. Gainor

**Affiliations:** 1 Department of Radiology, Massachusetts General Hospital, Boston, MA 02114, USA; 2 Massachusetts General Hospital Cancer Center and Department of Medicine, Massachusetts General Hospital, Boston, MA 02114, USA

**Keywords:** lung cancer, *RET* rearrangement, mutation, radiology

## Abstract

Rearranged during transfection proto-oncogene (*RET*) fusions represent a potentially targetable oncogenic driver in non-small cell lung cancer (NSCLC). Imaging features and metastatic patterns of advanced *RET* fusion-positive (*RET*+) NSCLC are not well established. Our goal was to compare the imaging features and patterns of metastases in *RET*+, *ALK*+ and *ROS1*+ NSCLC. Patients with *RET*+, *ALK*+, or *ROS1*+ NSCLC seen at our institution between January 2014 and December 2018 with available pre-treatment imaging were identified. The clinicopathologic features, imaging characteristics, and the distribution of metastases were reviewed and compared. We identified 215 patients with NSCLC harboring *RET*, *ALK*, or *ROS1* gene fusion (*RET* = 32; *ALK* = 116; *ROS1* = 67). Patients with *RET*+ NSCLC were older at presentation compared to *ALK*+ and *ROS1*+ patients (median age: *RET* = 64 years; *ALK* = 51 years, *p* < 0.001; ROS = 54 years, *p* = 0.042) and had a higher frequency of neuroendocrine histology (*RET* = 12%; *ALK* = 2%, *p* = 0.025; *ROS1* = 0%, *p* = 0.010). Primary tumors in *RET*+ patients were more likely to be peripheral (*RET* = 69%; *ALK* = 47%, *p* = 0.029; *ROS1* = 36%, *p* = 0.003), whereas lobar location, size, and density were comparable across the three groups. *RET*+ NSCLC was associated with a higher frequency of brain metastases at diagnosis compared to *ROS1*+ NSCLC (*RET* = 32%, *ROS1* = 10%; *p* = 0.039. Metastatic patterns were otherwise similar across the three molecular subgroups, with high incidences of lymphangitic carcinomatosis, pleural metastases, and sclerotic bone metastases. *RET*+ NSCLC shares several distinct radiologic features and metastatic spread with *ALK*+ and *ROS1+* NSCLC. These features may suggest the presence of *RET* fusions and help identify patients who may benefit from further molecular genotyping.

## 1. Introduction

Chromosomal rearrangements are an important class of driver oncogene alterations in non-small cell lung cancer (NSCLC). Validated examples of oncogenic fusion drivers in NSCLC include anaplastic lymphoma kinase (*ALK*) and *ROS1* gene fusions [1,2,3,4]. Advanced NSCLC harboring either *ALK* or *ROS1* fusion can be effectively treated with tyrosine kinase inhibitors (TKIs) targeting *ALK* or *ROS1*, respectively, and several *ALK*/*ROS1* tyrosine kinase inhibitors (TKIs) are now approved by the United States Food and Drug Administration.

Fusions involving the rearranged during transfection proto-oncogene (*RET*) occur in 1–2% of NSCLC [5,6,7], and are more commonly seen in patients with minimal to no smoking history and adenocarcinoma histologic subtype, reminiscent of *ALK* and *ROS1* fusions [8,9,10,11]. *RET* fusions are mutually exclusive with other oncogenic drivers such as *ALK* and *ROS1* fusions. Until recently, *RET*-selective TKIs were not available, and multitargeted TKIs with anti-*RET* activity such as cabozantinib and vandetanib were therefore tested in patients with advanced *RET* fusion-positive (*RET*+) NSCLC, with modest activity and significant toxicities [12,13,14,15,16,17,18,19,20,21]. More recently, two highly potent, *RET*-selective TKIs, BLU-667 and LOXO-292, have entered the clinic (ClinicalTrials.gov identifier NCT03037385 and NCT03157128, respectively). Both of these agents have demonstrated promising preliminary safety and efficacy profiles in patients with advanced solid tumors harboring *RET* alterations, including *RET*+ NSCLC [15,22,23,24,25].

Prior studies have suggested distinct imaging features that may be seen in NSCLCs harboring specific driver mutations, including *ALK* and *ROS1* fusions [26,27,28,29,30,31,32,33,34,35]. Less is known, however, regarding the radiologic features of advanced *RET*+ NSCLC. Here, we performed a retrospective analysis to determine the imaging features and metastatic patterns of advanced *RET+* NSCLC, specifically as compared to those of *ALK*+ and *ROS1*+ NSCLC, the most common oncogenic fusions in lung cancer.

## 2. Results

### 2.1. Patient Characteristics

The study included 215 patients with advanced NSCLC harboring either *RET*, *ALK*, or *ROS1* gene fusions (*RET*: 32; *ALK*: 116; *ROS1*: 67). Baseline characteristics of these patients are summarized in Table 1. Patients with *RET+* NSCLC were older at initial diagnosis compared to those with *ALK+* or *ROS1+* NSCLC (median age: *RET*: 64 years vs *ALK*: 51 years, *p* < 0.001; vs *ROS1*: 54 years, *p* = 0.042). While the majority of tumors in all three molecular subgroups had adenocarcinoma histology, there was a higher frequency of neuroendocrine histology among *RET+* tumors included in this study (*RET*: 12% vs *ALK*: 2%, *p* = 0.025; vs *ROS1*: 0%, *p* = 0.010). A higher proportion of *RET*+ patients in this cohort presented initially with stage I-II disease (*RET*: 25% vs *ALK*: 6%, *p* = 0.004; vs *ROS1*: 7%, *p* = 0.023).

### 2.2. Imaging Features of the Primary Tumor

We first evaluated the radiologic features of the primary tumor in *RET*+ NSCLC compared to *ALK*+ or *ROS1*+ NSCLC. *RET+* primary tumors were more likely to be peripheral in location compared to *ALK*+ and *ROS1+* tumors (*RET*: 69% vs *ALK*: 47%, *p* = 0.029; vs *ROS1*: 36%, *p* = 0.003). Otherwise, no significant differences were observed among the three groups with respect to primary tumor size, density, or lobar location (Table 2). A vast majority of the primary tumors were solid in density. Air bronchograms, cavities, and calcifications were infrequently observed in all three subgroups without a significant difference.

### 2.3. Patterns of Metastases

We next compared patterns of metastases at initial diagnosis as validated by radiologic imaging among the three gene fusion groups, as summarized in Table 3. A higher frequency of brain metastasis was noted at initial diagnosis in *RET+* NSCLC compared to *ROS1+* NSCLC (*RET*: 32% vs *ROS1*: 10%; *p* = 0.039); however, the frequency of brain metastasis at initial diagnosis was not significantly different between *RET*+ and *ALK*+ NSCLC (*RET*: 32% vs *ALK*: 25%; *p* = 0.592). The incidences of lung, pleural, lymphangitic, or pericardial spread were comparable across the three molecular subgroups. All three fusion cohorts had a high incidence of extrathoracic metastases (detected in 59%–77% of patients), including to the bone, without a significant difference (Table 3). Sclerotic-type bone metastases were common in the three molecular subgroups (*RET*: 80% vs *ALK*: 68%, *p* = 0.703; vs *ROS1*: 56%; *p* = 0.399).

## 3. Discussion

*RET* fusions are a targetable oncogenic driver in NSCLC, with two promising *RET*-selective inhibitors now on the horizon [15,22,23,24]. To our knowledge, this is the largest study to date to systematically assess the imaging features of the primary tumor and patterns of distant metastases in advanced *RET+* NSCLC, in comparison to *ALK*+ and *ROS1*+ NSCLC. Our findings revealed that the primary tumors in advanced *RET*+ NSCLC tended to be solid in density and not typically associated with air bronchograms, cavitation, or calcification, similar to *ALK*+ and *ROS1*+ NSCLC. However, primary *RET*+ tumors were more likely to be peripheral rather than central in location. All three fusion subgroups were relatively frequently associated with pleural metastases and lymphangitic carcinomatosis and extrathoracic metastases, brain, and bone metastases, at initial presentation. When bone metastases were present, these tended to be sclerotic rather than lytic.

These findings add to the emerging literature evaluating radiologic features and metastatic patterns in tumors harboring specific oncogenic drivers. Several prior studies have reported that certain imaging features may suggest the presence of gene alterations in NSCLC, and that the presence of these radiologic features may therefore help with triaging patients for expedited or repeat molecular testing when clinically warranted [26,27,28,36,37,38,39,40]. The available data on the imaging features of *RET+* NSCLC are more limited [31,38,39]. While molecular testing of the more common targetable alterations, including *EGFR* mutations and *ALK* fusions, in advanced NSCLC is recommended by national and international lung cancer guidelines, testing for *RET* fusions has not yet been as widely adopted, at least partly owing to the dearth of selective *RET* inhibitors until recently [41,42]. In select patients with clinical and imaging features suggestive of *RET*+ NSCLC—and in the absence of other well-validated targets such as *EGFR* or *BRAF* mutations, *ALK* or *ROS1* fusions on initial genotyping—it would be prudent to consider further testing for *RET* fusions so as to potentially expand effective treatment options for these patients.

**Figure 1:**
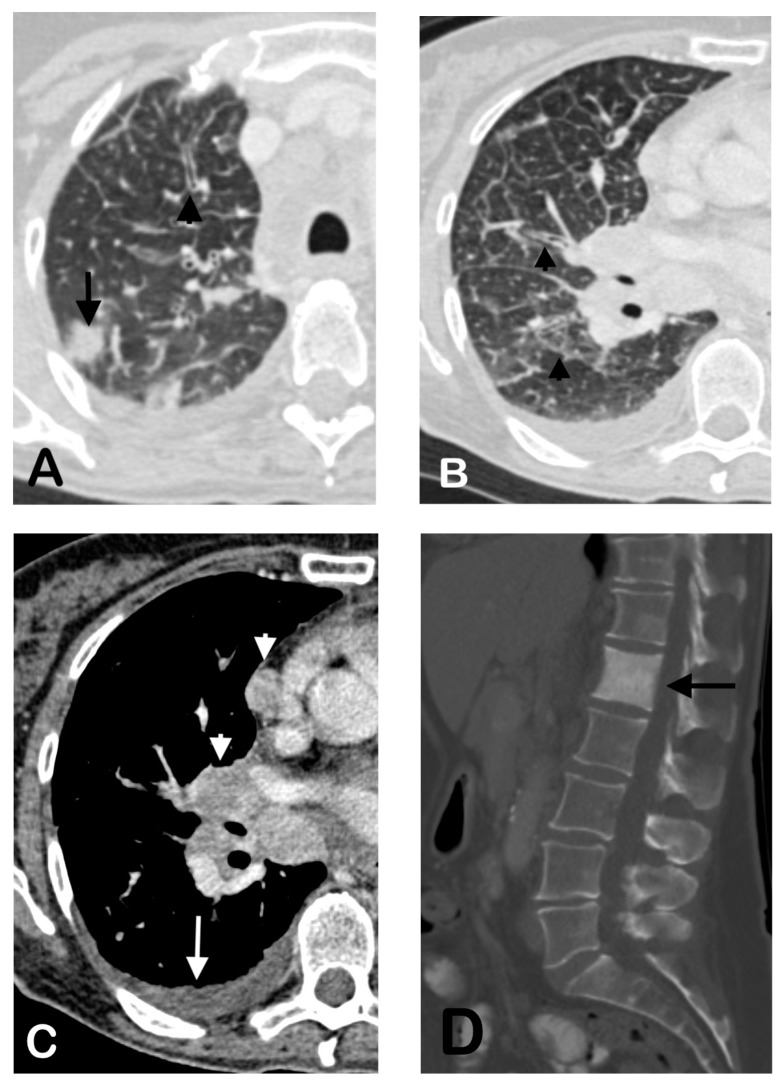
Representative imaging features in a 62-year-old female with *RET*+ NSCLC. Pretreatment CT shows a solid nodule in the peripheral right upper lobe (**A**, arrow) and associated septal and peribronchial thickening consistent with lymphangitic carcinomatosis (**A**,**B**, arrowheads). There was also an associated malignant pleural effusion (**C**, arrow), mediastinal and hilar lymphadenopathy (**C**, arrowheads)**,** and a sclerotic osseous metastasis of the first lumbar vertebral body (**D**, arrow).

In our cohort, the *RET*+ NSCLC primary tumors were mostly solid without associated air bronchograms, cavitation, or calcification (Figure 1). These primary tumor imaging features are not significantly different compared to the *ALK*+ and *ROS1*+ NSCLC tumors included in our cohort (Table 2) or compared to prior reports on *ALK* or *ROS1*+ tumors [26,31,37,38,43]. In this study, the primary *RET*+ tumors were more commonly peripheral rather than central in location, concordant with available literature [38,39]. Plodkowski et al. similarly reported that *RET+* NSCLC had an increased tendency for peripheral location [38]. However, their main comparison subgroup included *EGFR*-mutant NSCLC, rather than *ALK*+ NSCLC. Comparison with *ALK*+ NSCLC may be of added utility given the significant overlap in the clinical features of the two fusion driver subgroups [8,18,44,45]. An additional study by Saiki et al. also suggested that most primary tumors in *RET+* NSCLC were peripherally located [39]. The peripheral propensity of *RET*+ tumors may have potential diagnostic or therapeutic implications, such as approaches to tissue sampling, surgery, or the delivery of radiation [46,47,48].

The frequency of brain metastases at initial diagnosis of advanced *RET*+ NSCLC observed in our study is noteworthy. Approximately a third (32%) of patients with *RET*+ NSCLC were found to have baseline brain metastases at initial diagnosis, which is comparable to the incidence detected in *ALK*+ NSCLC and higher than that in *ROS1*+ NSCLC (Table 3). Previous studies have suggested that *ALK*+ NSCLC may be associated with a higher frequency of brain metastases at initial diagnosis and cumulatively [34], and that *RET*+ NSCLC may be associated with a significant cumulative incidence of brain metastases during patients’ disease course (at a level intermediate between that observed in *ALK*+ NSCLC and *ROS1*+ NSCLC) [35]. The significant incidence of brain metastases in *RET*+ disease underscores the importance of developing RET-targeted therapeutic agents with robust central nervous system (CNS) activity. In this regard, both *RET*-selective TKIs, BLU-667 and LOXO-292 have demonstrated preliminary encouraging CNS activity in patients with advanced *RET*+ NSCLC and baseline brain metastases [19,23,25,35].

The patients with *RET+* NSCLC in our cohort, otherwise, shared similar patterns of metastases to those with *ALK+* or *ROS1+* NSCLC, including high frequencies of pleural and bone metastases and lymphangitic carcinomatosis (Table 3; Figure 1). Notably, the osseous metastases in all three fusion subgroups tended to be predominantly sclerotic, rather than lytic as has previously been associated with NSCLC (Figure 1) [49,50]. To our knowledge, no prior studies have reported the propensity for sclerotic bone metastases in *RET*+. Notably, a similar predilection for sclerotic bone metastases has been reported in *ALK*+, or *ROS1+* NSCLC [29,40]. The molecular mechanisms underlying the propensity for the development of sclerotic osseous metastases in these fusion-driven lung cancers remain to be studied.

Our study had several limitations. Although this is the largest study to date to assess the imaging features and metastatic patterns in *RET+* NSCLC, our cohort is still relatively small due to the rarity of *RET* alterations in NSCLC overall. Thus, the statistical power was limited in determining significant differences among the three molecular subgroups. The moderate sample sizes also precluded multivariable analysis adjusting for confounding factors to obtain stable and precise estimates of any effect size. The data were collected retrospectively from a single institution, predisposing to selection and referral bias and potentially limiting their generalizability to larger populations. Additionally, the study did not evaluate for differences between *RET*+ NSCLC and oncogenic subsets of NSCLC other than *ALK*+ or *ROS1*+ NSCLC (such as *EGFR*-mutant NSCLC, *BRAF*-mutant NSCLC, etc.). These comparator cohorts were chosen because *ALK* and *ROS1* fusions represent two well-validated fusion targets in NSCLC. Despite these limitations, our findings add to the growing understanding of the clinical and radiologic features of *RET+* NSCLC. In select patients with clinical and imaging features suggestive of *RET*+ NSCLC, and negative testing for *EGFR*, *BRAF*, *ALK*, or *ROS1* alterations, further molecular testing for *RET* fusions may be advisable.

## 4. Materials and Methods

### 4.1. Patient Identification

This retrospective study was performed under an institutional review board-approved protocol (Partners Human Research Protocol #: 2019P000198). Patients were eligible if they fulfilled the following criteria: (1) had the diagnosis of advanced/metastatic NSCLC with a *RET, ALK,* or *ROS1* fusion confirmed by locally accepted testing at the time of presentation (e.g., fluorescent in situ hybridization, polymerase chain reaction, next-generation sequencing, and/or immunohistochemistry (for *ALK*)); (2) were seen in the thoracic oncology clinic of Massachusetts General Hospital between January 2014 and December 2018; and (3) had pre-treatment imaging performed at our institution, or at another facility, with the examination uploaded into the picture archiving and communication system (PACS) for radiologist review. The testing method used to detect the driver mutations varied depending on when the patient presented. Clinicopathologic data were extracted from the electronic medical records, including patient age, sex, race, smoking history, tumor histology, and stage of disease at initial diagnosis per the American Joint Committee on Cancer TNM Staging, 7th edition.

### 4.2. Imaging Review and Analysis

Baseline imaging data obtained at diagnosis prior to the initiation of therapy (e.g., surgery, radiation, or systemic therapy) were selected and reviewed on the institutional PACS (AGFA Impax 6, Mortsel, Belgium). Required imaging studies included, at minimum, computed tomography (CT) scans of the chest, abdomen, and pelvis with or without concurrent fluorodeoxyglucose (FDG)-positron emission tomography (PET), and CT and/or magnetic resonance imaging (MRI) of the brain. Radiologic findings were determined by consensus between two radiologists (S.R.D. and D.P.M.).

When a primary tumor was identifiable, CT features of the primary tumor were analyzed with regard to its size, lobe, axial location (i.e., central versus peripheral), density (i.e., solid versus subsolid), and the presence or absence of cavitation, air bronchograms, or calcification. Images were also assessed for lymphadenopathy and thickening of the interstitium suggestive of lymphangitic carcinomatosis. A lymph node was considered malignant if it measured greater than 1 cm in its short axis and/or had increased FDG uptake on 18-FDG PET imaging. Malignant lymph nodes were recorded as ipsilateral or contralateral, and as hilar, mediastinal, supraclavicular, or distant (e.g., cervical, axillary, intra-abdominal, etc.). Other sites examined for evidence of metastases included the lungs, pleura, pericardium, liver, adrenal glands, other visceral organs (e.g., spleen, kidney), brains, bones, and subcutaneous soft tissues. Bone metastases were additionally classified as predominantly lytic versus predominantly blastic or sclerotic.

### 4.3. Statistical Analysis

To preserve power given modest sample sizes, we performed pairwise comparisons between patients with *RET*+ NSCLC and patients with *ALK*+ or *ROS1*+ NSCLC. Fisher’s exact test was used to compare clinicopathologic characteristics of patients and imaging features of the primary tumor between molecular subgroups as well as distribution of metastatic sites among patients with advanced NSCLC. Wilcoxon rank-sum test was used to analyze the age distribution at initial diagnosis. Data analysis was performed using SAS 9.4 (SAS Institute Inc., Cary, NC, USA), and *p*-values were reported for two-sided tests.

## 5. Conclusions

*RET*+ NSCLC shares several radiologic features with *ALK*+ and *ROS1+* NSCLC, including solid density of the primary tumor and high frequencies of lymphangitic carcinomatosis and pleural, brain, and bone metastases. When bone metastases are present, these are often sclerotic in nature. These imaging features may suggest the presence of *RET* fusions and help triage those patients who may benefit from further molecular genotyping.

## Figures and Tables

**Table 1:** Clinicopathologic features of patients with *RET*, *ALK*, and *ROS1* fusion-positive NSCLC.

Clinical Feature	*RET*	*ALK*	*ROS1*	*RET* vs. *ALK*	*RET* vs. *ROS1*
	(*N* = 32)	(*N* = 116)	(*N* = 67)	*p*-Value	*p*-Value
**Age**					
Median	64	51	54	*<0.0001*	*0.042*
Range	(42–83)	(19–75)	(23–89)		
**Sex**					
Female	14 (44%)	66 (57%)	49 (73%)	0.230	*0.007*
Male	18 (56%)	50 (43%)	18 (27%)		
**Race**					
Caucasian	26 (81%)	90 (78%)	49 (73%)	0.868	0.378
Asian	5 (16%)	17 (15%)	10 (15%)		
Other	1 (3%)	9 (8%)	8 (12%)		
**Smoking history**					
Never	23 (72%)	87 (75%)	45 (67%)	0.820	0.817
Prior/current	9 (28%)	29 (25%)	22 (33%)		
**Tumor histology**					
Adenocarcinoma	28 (88%)	108 (93%)	67 (100%)	*0.025*	*0.010*
Neuroendocrine	4 (12%)	2 (2%)	0		
Other	0	6 (5%)	0		
**Stage**					
I-II	8 (25%)	7 (6%)	5 (7%)	*0.0044*	*0.023*
III	2 (6%)	22 (19%)	13 (19%)		
IV	22 (69%)	87 (75%)	49 (73%)		

Statistically significant *p*-values are highlighted.

**Table 2:** Imaging features of the primary tumor in patients with *RET*, *ALK*, or *ROS1* fusion-positive NSCLC.

Imaging Feature	*RET*	*ALK*	*ROS1*	*RET* vs. *ALK*	*RET* vs. *ROS1*
	(*N* = 32)	(*N* = 116)	(*N* = 67)	*p*-Value	*p*-Value
Tumor size (mm)					
Median	32	41	33	0.258	0.836
Range	(9–89)	(5–115)	(10–126)		
≥3 cm	19 (59%)	80 (69%)	37 (55%)	0.396	0.829
Density					
Solid	30 (94%)	115 (99%)	64 (96%)	0.118	0.657
Subsolid	2 (6%)	1 (1%)	3 (4%)		
Other features					
Air bronchograms	2 (6%)	7 (6%)	3 (4%)	1.000	0.657
Cavitation	0	7 (6%)	2 (3%)	0.347	1.000
Calcification	0	0	0		
Tumor location					
RUL	4 (12%)	28 (24%)	14 (21%)	0.249	0.477
RML	6 (19%)	10 (9%)	10 (15%)		
RLL	8 (25%)	24 (21%)	19 (28%)		
LUL	8 (25%)	22 (19%)	8 (12%)		
LLL	6 (19%)	32 (28%)	16 (24%)		
Lobar level					
Lower	14 (44%)	56 (48%)	35 (52%)	0.693	0.521
Upper	18 (56%)	60 (52%)	32 (48%)		
Laterality					
Left	14 (44%)	54 (47%)	24 (36%)	0.843	0.510
Right	18 (56%)	62 (53%)	43 (64%)		
Axial location					
Central	10 (31%)	62 (53%)	43 (64%)	*0.029*	*0.003*
Peripheral	22 (69%)	54 (47%)	24 (36%)		

RUL = right upper lobe; RML = right middle lobe; RLL = right lower lobe; LUL = left upper lobe; LLL = left lower lobe. Statistically significant *p*-values are highlighted.

**Table 3:** Patterns of metastatic sites among patients with advanced *RET*, *ALK*, or *ROS1* fusion-positive NSCLC.

Metastatic Site	*RET*	*ALK*	*ROS1*	*RET* vs. *ALK*	*RET* vs. *ROS1*
	(*N* = 22)	(*N* = 87)	(*N* = 49)	*p*-Value	*p*-Value
**Intrathoracic**	14 (64%)	64 (74%)	41 (84%)	0.429	0.074
Lung	4 (18%)	18 (21%)	18 (37%)	1.000	0.167
Pleural	10 (45%)	35 (40%)	20 (41%)	0.809	0.797
Lymphangitic carcinomatosis	6 (27%)	35 (40%)	21 (43%)	0.329	0.292
Pericardium	1 (5%)	2 (2%)	2 (4%)	0.495	1.000
**Extrathoracic**	17 (77%)	65 (75%)	29 (59%)	1.000	0.183
Bone	10 (45%)	41 (47%)	16 (33%)	1.000	0.425
Sclerotic metastasis	8 (80%)	28 (68%)	9 (56%)	0.703	0.399
Liver	3 (14%)	21 (24%)	10 (20%)	0.393	0.741
Brain	7 (32%)	22 (25%)	5 (10%)	0.592	*0.039*
Distant lymph nodes	5 (23%)	17 (20%)	8 (16%)	0.769	0.524
Adrenal	4 (18%)	6 (7%)	7 (14%)	0.114	0.729
Soft tissue	1 (5%)	5 (6%)	1 (2%)	1.000	0.527

## References

[1] Rikova K., Guo A., Zeng Q., Possemato A., Yu J., Haack H., Nardone J., Lee K., Reeves C., Li Y. (2007). Global survey of phosphotyrosine signaling identifies oncogenic kinases in lung cancer. Cell.

[2] Soda M., Choi Y.L., Enomoto M., Takada S., Yamashita Y., Ishikawa S., Fujiwara S., Watanabe H., Kurashina K., Hatanaka H. (2007). Identification of the transforming EML4-ALK fusion gene in non-small-cell lung cancer. Nature.

[3] Lin J.J., Shaw A.T. (2017). Recent advances in targeting ROS1 in lung cancer. J. Thorac. Oncol..

[4] Lin J.J., Riely G.J., Shaw A.T. (2017). Targeting ALK: Precision medicine takes on drug resistance. Cancer Discov..

[5] Ju Y.S., Lee W.-C., Shin J.-Y., Lee S., Bleazard T., Won J.-K., Kim Y.T., Kim J.-I., Kang J.-H., Seo J.-S. (2012). A transforming KIF5B and RET gene fusion in lung adenocarcinoma revealed from whole-genome and transcriptome sequencing. Genome Res..

[6] Kohno T., Ichikawa H., Totoki Y., Yasuda K., Hiramoto M., Nammo T., Sakamoto H., Tsuta K., Furuta K., Shimada Y. (2012). KIF5B-RET fusions in lung adenocarcinoma. Nat. Med..

[7] Lipson D., Capelletti M., Yelensky R., Otto G., Parker A., Jarosz M., Curran J.A., Balasubramanian S., Bloom T., Brennan K.W. (2012). Identification of new ALK and RET gene fusions from colorectal and lung cancer biopsies. Nat. Med..

[8] Wang R., Hu H., Pan Y., Li Y., Ye T., Li C., Luo X., Wang L., Li H., Zhang Y. (2012). RET fusions define a unique molecular and clinicopathologic subtype of non-small-cell lung cancer. J. Clin. Oncol..

[9] Bergethon K., Shaw A.T., Ignatius Ou S.-H., Katayama R., Lovly C.M., McDonald N.T., Massion P.P., Siwak-Tapp C., Gonzalez A., Fang R. (2012). ROS1 Rearrangements define a unique molecular class of lung cancers. J. Clin. Oncol..

[10] Gautschi O., Milia J., Filleron T., Wolf J., Carbone D.P., Owen D., Camidge R., Narayanan V., Doebele R.C., Besse B. (2017). Targeting RET in patients with RET-rearranged lung cancers: Results from the global, multicenter RET registry. J. Clin. Oncol..

[11] Shaw A.T., Yeap B.Y., Mino-Kenudson M., Digumarthy S.R., Costa D.B., Heist R.S., Solomon B., Stubbs H., Admane S., McDermott U. (2009). Clinical features and outcome of patients with non-small-cell lung cancer who harbor EML4-ALK. J. Clin. Oncol..

[12] Falchook G.S., Ordóñez N.G., Bastida C.C., Stephens P.J., Miller V.A., Gaido L., Jackson T., Karp D.D. (2016). Effect of the RET inhibitor vandetanib in a patient with RET fusion-positive metastatic non-small-cell lung cancer. J. Clin. Oncol..

[13] Li G.G., Somwar R., Joseph J., Smith R.S., Hayashi T., Martin L., Franovic A., Schairer A., Martin E., Riely G.J. (2017). Antitumor activity of RXDX-105 in multiple cancer types with RET rearrangements or mutations. Clin. Cancer Res..

[14] Takeuchi S., Murayama T., Yoshimura K., Kawakami T., Takahara S., Imai Y., Kuribayashi Y., Nagase K., Goto K., Nishio M. (2017). Phase I/II study of alectinib in lung cancer with RET fusion gene: Study protocol. J. Med. Investig..

[15] Subbiah V., Velcheti V., Tuch B.B., Ebata K., Busaidy N.L., Cabanillas M.E., Wirth L.J., Stock S., Smith S., Lauriault V. (2018). Selective RET kinase inhibition for patients with RET-altered cancers. Ann. Oncol..

[16] Drilon A., Rekhtman N., Arcila M., Wang L., Ni A., Albano M., Van Voorthuysen M., Somwar R., Smith R.S., Montecalvo J. (2016). Cabozantinib in patients with advanced RET-rearranged non-small-cell lung cancer: An open-label, single-centre, phase 2, single-arm trial. Lancet Oncol..

[17] Horiike A., Takeuchi K., Uenami T., Kawano Y., Tanimoto A., Kaburaki K., Tambo Y., Kudo K., Yanagitani N., Ohyanagi F. (2016). Sorafenib treatment for patients with RET fusion-positive non-small cell lung cancer. Lung Cancer.

[18] Lin J.J., Kennedy E., Sequist L.V., Brastianos P.K., Goodwin K.E., Stevens S., Wanat A.C., Stober L.L., Digumarthy S.R., Engelman J.A. (2016). Clinical activity of alectinib in advanced RET-rearranged non-small cell lung cancer. J. Thorac. Oncol..

[19] Drilon A., Fu S., Patel M.R., Fakih M., Wang D., Olszanski A.J., Morgensztern D., Liu S.V., Cho B.C., Bazhenova L. (2019). A phase I/Ib trial of the VEGFR-sparing multikinase RET inhibitor RXDX-105. Cancer Discov..

[20] Lee S.-H., Lee J.-K., Ahn M.-J., Kim D.-W., Sun J.-M., Keam B., Kim T.M., Heo D.S., Ahn J.S., Choi Y.-L. (2017). Vandetanib in pretreated patients with advanced non-small cell lung cancer-harboring RET rearrangement: A phase II clinical trial. Ann. Oncol..

[21] Yoh K., Seto T., Satouchi M., Nishio M., Yamamoto N., Murakami H., Nogami N., Matsumoto S., Kohno T., Tsuta K. (2017). Vandetanib in patients with previously treated RET-rearranged advanced non-small-cell lung cancer (LURET): An open-label, multicentre phase 2 trial. Lancet Respir. Med..

[22] Subbiah V., Gainor J.F., Rahal R., Brubaker J.D., Kim J.L., Maynard M., Hu W., Cao Q., Sheets M.P., Wilson D. (2018). Precision targeted therapy with BLU-667 for RET-driven cancers. Cancer Discov..

[23] Gainor J.F., Lee D.H., Curigliano G., Doebele R.C., Kim D.-W., Baik C.S., Tan D.S.-W., Lopes G., Gadgeel S.M., Cassier P.A. (2019). Clinical activity and tolerability of BLU-667, a highly potent and selective RET inhibitor, in patients (pts) with advanced RET-fusion+ non-small cell lung cancer (NSCLC). J. Clin. Oncol..

[24] Subbiah V., Taylor M., Lin J., Hu M., Ou S.-H.I., Brose M.S., Garralda E., Clifford C., Palmer M., Evans E. (2018). Abstract CT043: Highly potent and selective RET inhibitor, BLU-667, achieves proof of concept in a phase I study of advanced, RET-altered solid tumors. Cancer Res..

[25] Drilon A.E., Subbiah V., Oxnard G.R., Bauer T.M., Velcheti V., Lakhani N., Besse B., Park K., Patel J.D., Cabanillas M.E. (2018). A phase 1 study of LOXO-292, a potent and highly selective RET inhibitor, in patients with RET-altered cancers. J. Clin. Oncol..

[26] Mendoza D.P., Dagogo-Jack I., Chen T., Padole A., Shepard J.-A.O., Shaw A.T., Digumarthy S.R. (2019). Imaging characteristics of BRAF-mutant non-small cell lung cancer by functional class. Lung Cancer.

[27] Mendoza D.P., Stowell J., Muzikansky A., Shepard J.-A.O., Shaw A.T., Digumarthy S.R. (2019). Computed tomography imaging characteristics of non-small-cell lung cancer with anaplastic lymphoma kinase rearrangements: A systematic review and meta-analysis. Clin. Lung Cancer.

[28] Digumarthy S.R., Mendoza D.P., Padole A., Chen T., Peterson P.G., Piotrowska Z., Sequist L.V. (2019). Diffuse lung metastases in EGFR-mutant non-small cell lung cancer. Cancers.

[29] Mendoza D.P., Lin J.J., Rooney M.M., Chen T., Sequist L.V., Shaw A.T., Digumarthy S.R. (2019). Imaging features and metastatic patterns of advanced ALK-rearranged non-small cell lung cancer. Am. J. Roentgenol..

[30] Digumarthy S.R., Mendoza D.P., Zhang E.W., Lennerz J.K., Heist R.S. (2019). Clinicopathologic and imaging features of non-small-cell lung cancer with MET exon 14 skipping mutations. Cancers.

[31] Yoon H.J., Sohn I., Cho J.H., Lee H.Y., Kim J.-H., Choi Y.-L., Kim H., Lee G., Lee K.S., Kim J. (2015). Decoding tumor phenotypes for ALK, ROS1, and RET fusions in lung adenocarcinoma using a radiomics approach. Medicine (Baltimore).

[32] Fukui T., Yatabe Y., Kobayashi Y., Tomizawa K., Ito S., Hatooka S., Matsuo K., Mitsudomi T. (2012). Clinicoradiologic characteristics of patients with lung adenocarcinoma harboring EML4-ALK fusion oncogene. Lung Cancer.

[33] Park J., Kobayashi Y., Urayama K.Y., Yamaura H., Yatabe Y., Hida T. (2016). Imaging characteristics of driver mutations in EGFR, KRAS, and ALK among treatment-naïve patients with advanced lung adenocarcinoma. PLoS ONE.

[34] Gainor J.F., Tseng D., Yoda S., Dagogo-Jack I., Friboulet L., Lin J.J., Hubbeling H.G., Dardaei L., Farago A.F., Schultz K.R. (2017). Patterns of metastatic spread and mechanisms of resistance to crizotinib in ROS1-positive non–small-cell lung cancer. JCO Precis. Oncol..

[35] Drilon A., Lin J.J., Filleron T., Ni A., Milia J., Bergagnini I., Hatzoglou V., Velcheti V., Offin M., Li B. (2018). Frequency of brain metastases and multikinase inhibitor outcomes in patients with RET-rearranged lung cancers. J. Thorac. Oncol..

[36] Digumarthy S.R., Padole A.M., Gullo R.L., Sequist L.V., Kalra M.K. (2019). Can CT radiomic analysis in NSCLC predict histology and EGFR mutation status?. Medicine (Baltimore).

[37] Yamamoto S., Korn R.L., Oklu R., Migdal C., Gotway M.B., Weiss G.J., Iafrate A.J., Kim D.-W., Kuo M.D. (2014). ALK molecular phenotype in non-small cell lung cancer: CT radiogenomic characterization. Radiology.

[38] Plodkowski A.J., Drilon A., Halpenny D.F., O’Driscoll D., Blair D., Litvak A.M., Zheng J., Moskowitz C.S., Ginsberg M.S. (2015). From genotype to phenotype: Are there imaging characteristics associated with lung adenocarcinomas harboring RET and ROS1 rearrangements?. Lung Cancer.

[39] Saiki M., Kitazono S., Yoshizawa T., Dotsu Y., Ariyasu R., Koyama J., Sonoda T., Uchibori K., Nishikawa S., Yanagitani N. (2018). Characterization of computed tomography imaging of rearranged during transfection-rearranged lung cancer. Clin. Lung Cancer.

[40] Digumarthy S.R., Mendoza D.P., Lin J.J., Chen T., Rooney M.M., Chin E., Sequist L.V., Lennerz J.K., Gainor J.F., Shaw A.T. (2020). Computed tomography imaging features and distribution of metastases in ROS1-rearranged non-small-cell lung cancer. Clin. Lung Cancer.

[41] Planchard D., Popat S., Kerr K., Novello S., Smit E.F., Faivre-Finn C., Mok T.S., Reck M., Van Schil P.E., Hellmann M.D. (2018). Metastatic non-small cell lung cancer: ESMO Clinical Practice Guidelines for diagnosis, treatment and follow-up. Ann. Oncol..

[42] Ettinger D.S., Aisner D.L., Wood D.E., Akerley W., Bauman J., Chang J.Y., Chirieac L.R., D’Amico T.A., Dilling T.J., Dobelbower M. (2018). NCCN guidelines insights: Non-small cell lung cancer, version 5.2018. J. Natl. Compr. Cancer Netw..

[43] Halpenny D.F., Riely G.J., Hayes S., Yu H., Zheng J., Moskowitz C.S., Ginsberg M.S. (2014). Are there imaging characteristics associated with lung adenocarcinomas harboring ALK rearrangements?. Lung Cancer.

[44] Gainor J.F., Shaw A.T. (2013). Novel targets in non-small cell lung cancer: ROS1 and RET fusions. Oncologist.

[45] Kodama T., Tsukaguchi T., Satoh Y., Yoshida M., Watanabe Y., Kondoh O., Sakamoto H. (2014). Alectinib shows potent antitumor activity against RET-rearranged non-small cell lung cancer. Mol. Cancer Ther..

[46] Manhire A., Charig M., Clelland C., Gleeson F., Miller R., Moss H., Pointon K., Richardson C., Sawicka E. (2003). BTS Guidelines for radiologically guided lung biopsy. Thorax.

[47] British Thoracic Society Society of Cardiothoracic Surgeons of Great Britain Ireland Working Party (2001). Guidelines on the selection of patients with lung cancer for surgery. Thorax.

[48] Román A., Perez-Rozos A., Otero A., Jodar C., García-Ríos I., Lupiañez-Perez Y., Antonio Medina J., Gomez-Millan J. (2019). Efficacy and safety of a simplified SBRT regimen for central and peripheral lung tumours. Clin. Transl. Oncol..

[49] Ali Mohammed Hammamy R., Farooqui K., Ghadban W. (2018). Sclerotic bone metastasis in pulmonary adenocarcinoma. Case Rep. Med..

[50] Haghighatkhah H.R., Sanei Taheri M., Kharrazi S.M.H., Ghazanfari Amlashi D., Haddadi M., Pourabdollah M. (2012). An unusual case of pulmonary adenocarcinoma with multiple and extraordinary metastases. Iran. J. Radiol..

